# Evolution and New Horizons of Endoscopy in Inflammatory Bowel Diseases

**DOI:** 10.3390/jcm11030872

**Published:** 2022-02-07

**Authors:** Tommaso Lorenzo Parigi, Elisabetta Mastrorocco, Leonardo Da Rio, Mariangela Allocca, Ferdinando D’Amico, Alessandra Zilli, Gionata Fiorino, Silvio Danese, Federica Furfaro

**Affiliations:** 1 Department of Biomedical Sciences, Humanitas University, 20090 Milan, Italy; tommaso.parigi@humanitas.it (T.L.P.); elisabetta.mastrorocco@humanitas.it (E.M.); leonardo.dario@humanitas.it (L.D.R.); 2 Institute of Immunology and Immunotherapy, University of Birmingham, Birmingham B15 2TT, UK; 3 Gastroenterology and Endoscopy, IRCCS Ospedale San Raffaele, Vita-Salute San Raffaele University, 20132 Milan, Italy; mariangela.allocca@hsr.it (M.A.); ferdinando.damico@hsr.it (F.D.); alessandra.zilli@hsr.it (A.Z.); gionata.fiorino@hsr.it (G.F.); silvio.danese@hsr.it (S.D.); 4 IBD Center, Humanitas Research Hospital, 20089 Milan, Italy

**Keywords:** endoscopy, inflammatory bowel disease, ulcerative colitis, Crohn’s disease, artificial intelligence, virtual chromoendoscopy, capsule endoscopy, endocytoscopy, confocal laser endomicroscopy, molecular endoscopy

## Abstract

Endoscopy is the mainstay of inflammatory bowel disease (IBD) evaluation and the pillar of colorectal cancer surveillance. Endoscopic equipment, both hardware and software, are advancing at an incredible pace. Virtual chromoendoscopy is now widely available, allowing the detection of subtle inflammatory changes, thus reducing the gap between endoscopic and histologic assessment. The progress in the field of artificial intelligence (AI) has been remarkable, and numerous applications are now in an advanced stage of development. Computer-aided diagnosis (CAD) systems are likely to reshape most of the evaluations that are now prerogative of human endoscopists. Furthermore, sophisticated tools such as endocytoscopy and probe-based confocal laser endomicroscopy (pCLE) are enhancing our assessment of inflammation and dysplasia. Finally, pCLE combined with molecular labeling could pave the way to a new paradigm of personalized medicine. This review aims to summarize the main changes that occurred in the field of IBD endoscopy and to explore the most promising novelties.

## 1. Introduction

Inflammatory bowel diseases (IBD) are a group of immune-mediated conditions of the gastrointestinal tract, associated with significant morbidity and reduction in quality of life. The two main types of IBD are Crohn’s disease (CD) and ulcerative colitis (UC). The role of endos in the management of IBD has gained importance over the past two decades. Coupled with histology, endoscopy is fundamental for diagnosis [1] and for the follow-up, allowing practitioners to assess the presence of disease activity or remission. Finally, endoscopy is also the cornerstone of CRC surveillance [1]. In all these instances, the gastroenterologist evaluates macroscopically the mucosa and collects random or targeted histological samples according to the clinical purpose. Novel techniques, presented below, expand the role of conventional endoscopy and bridge it to histology.

In the follow up of IBD, symptoms are not reliable indicators of the underlying inflammatory state. Several studies show a mismatch between the clinical presentation and objective measures of inflammation, such as fecal calprotectin and endoscopic activity [2,3]. The presence of mucosal inflammation is what ultimately correlates with complications such as flare up, surgery and hospitalization [4,5]. Therefore, the goal of treatment is to resolve the inflammation, rather than only the symptoms. Current guidelines, including the recently published STRIDE II consensus [6], recommend optimizing treatment aiming for endoscopic remission. Even more ambitious targets, such as transmural healing in CD and histological remission in UC [6], have been proposed and are currently considered important adjunctive measures, though not formally endorsed as treatment targets. In UC, the concept of mucosal healing (MH) has been proposed to encompass both endoscopic and histologic remission, whereas, in CD, the transmural nature of the disease complicates the definition of MH.

**Figure 1:**
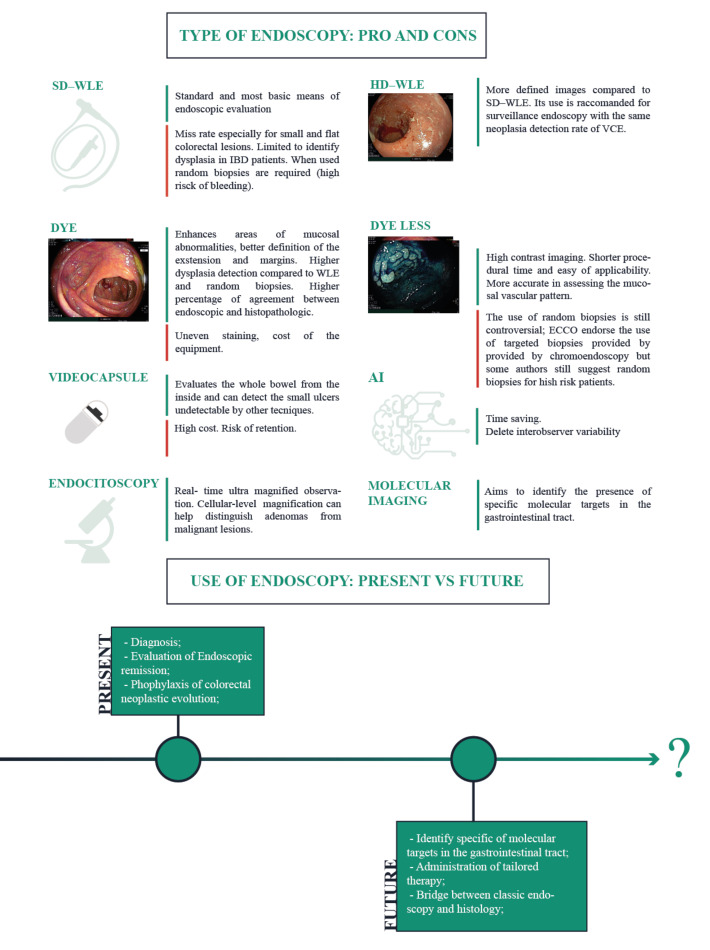
Evolution and new horizons of endoscopy in IBD (SD-WLE: Standard definition white light endoscopy; WLE-HD: High definition white light endoscopy; AI: artificial intelligence; VCE: virtual chromoendoscopy) [1,7,8,9,10,11,12,13,14,15,16,17,18,19,20].

Technological advances are reshaping the role of endoscopy and expanding it into other fields such as precision medicine. The aim of this review is to present the applications of endoscopy in the management of IBD and evaluate the latest advances and future developments (Figure 1).

## 2. Methods

A literature review was independently performed by three authors (T.L.P., E.M., L.D.R.) in the PubMed/MEDLINE databases up to December 2021 using the following search terms: “endoscopy”, “virtual chromoendoscopy”, “capsule endoscopy”, “artificial intelligence”, “machine learning”, “molecular endoscopy”, “molecular imaging” individually or in combination with “IBD”, “inflammatory bowel disease(s)”, “ulcerative colitis” or “Crohn’s disease”. Additionally, the abstracts presented at the ECCO congress, DDW and ESGE in 2021 were manually searched. The search focused on full-text papers published in English. Abstracts were selected when relevant. No publication date restrictions were imposed. Finally, articles were included in this review on the basis of their relevance, while additional publications were identified through their reference lists.

## 3. Endoscopy in IBD: Applications

### 3.1. Endoscopy Activity: Endoscopic Scores

#### 3.1.1. Endoscopic Scores for Crohn’s Disease

**Figure 2:**
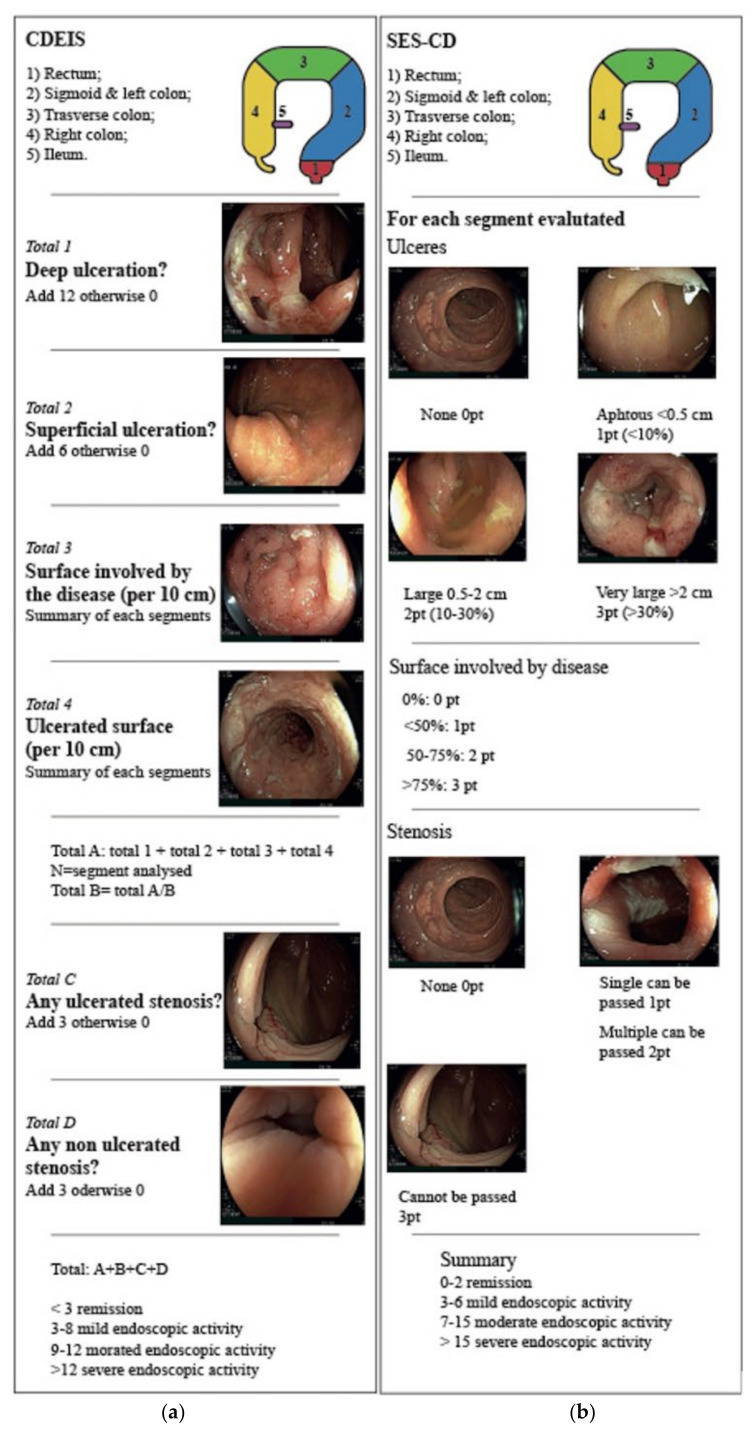
Endoscopic scores for Crohn’s disease: (**a**) CDEIS; (**b**) SES-CD.

The two main endoscopic scores validated for CD are the Crohn’s Disease Index of Severity (CDEIS) and the Simple Endoscopic Score for Crohn’s Disease (SES-CD) (Figure 2) [21,22]. CDEIS evaluates five segments of the intestine: rectum, sigmoid, left, traverse and right colon and ileum. In each segment, the endoscopist records the presence of deep or superficial ulcers, the percentage of surface ulcerated or involved by disease and the presence of ulcerated or non-ulcerated stenosis. A score is assigned for each element and summed to a total ranging from 0 (not active disease) to 44 (severe disease) [21]. Similarly, the SES-CD evaluates four endoscopic items (ulcer size, percentage of ulcerated surface, proportion of the surface area affected by disease and the presence of stenosis) in the same five anatomical locations. Each element is weighted, and a score is assigned from 0 to 56 [22].

In addition, as CD patients often undergo ileo-colonic resection, an ad hoc endoscopy score, Rutgeerts’ score, is used to predict the recurrence of disease (Table 1) [23].

Overall, the grading of CD activity is more challenging compared with UC for a number of reasons. The mucosal damage is usually discontinuous, hence the need to consider the location in the score, and transmural, whereas endoscopy is inherently limited to the mucosal surface. Moreover, different disease phenotypes exist: inflammatory, stenotic and fistulizing.

#### 3.1.2. Endoscopic Scores for Ulcerative Colitis

**Figure 3:**
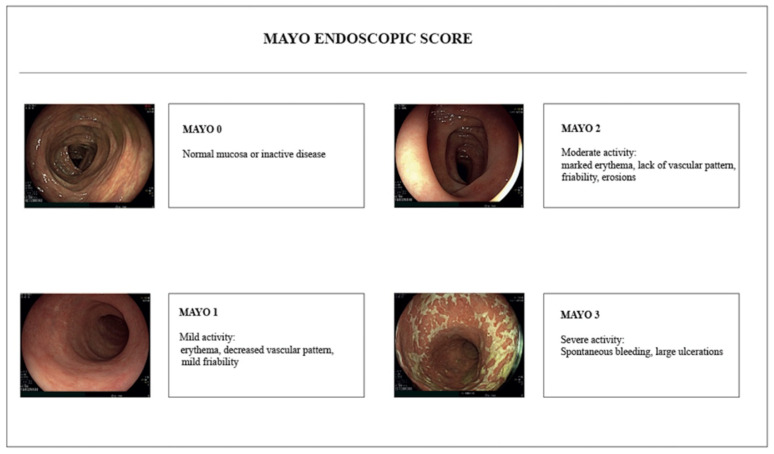
Mayo endoscopic score (MES).

Numerous scores have been proposed to assess UC endoscopic activity [24]. The Mayo endoscopic score (MES) (Figure 3), ranging from zero, normal mucosa, to three, severely active UC, is the most widely adopted. MES requires minimal training and is easily remembered; however, it lacks a formal validation and has been criticized for its subjective evaluation of findings such as erythema and friability [25]. Several other scores such as the Ulcerative Colitis Endoscopic Index of Severity (UCEIS) (Table 2) and the Ulcerative Colitis Colonoscopic Index (UCCIS) (Table 3) have been proposed to overcome some of the MES’s limitations [24]. In particular, both UCEIS and UCCIS, thanks to a more detailed objective description of findings, have stronger correlation with histology and lower inter-rater variability [26,27]. Finally, taking advantage of developments in virtual chromoendoscopy, the PICaSSO score has recently been shown to have the highest correlation with histology among endoscopic scores [28,29].

### 3.2. Surveillance Evolution

Patients with IBD have an increased risk of developing colorectal cancer (CRC). Consistently with the inflammation–dysplasia–cancer sequence, the risk is proportional to the duration and extension of disease [30]. In particular, during the first 10 years, the incidence rate is approximately 2/1000 patient years (pyd), and it triples in the second decade (7/1000 pyd) and increases to 12/1000 pyd in the third decade [31]. In other terms, the cumulative risk is around 2% after the first 10 years, 8% after 20 and 18% after 30 [31]. To mitigate the risk of CRC and diagnose precursor lesions or early cancer, patients with IBD undergo regular endoscopic surveillance. Because cancer incidence increases over time, surveillance should start 8 to 10 years after the initial diagnosis. Consistently with the hypothesis of inflammation driving cancer, the degree of disease activity correlates with the risk of malignancy [30]. One study classifying patients according to the severity of ongoing inflammation found a positive correlation between mucosal inflammation and incidence of neoplasia (hazard ratio [HR] ranging from 2.2 to 3.4; CI 1.2–4.2 and 1.1–10.4, respectively) [30]. In addition, other factors such as family history of CRC, concomitant primary sclerosing cholangitis or stricturing CD phenotype contribute to the risk and warrant closer surveillance. The time interval is established on the presence of risk factors and ranges from yearly colonoscopy in UC patients with active pancolitis or PSC to five yearly colonoscopies for left-sided quiescent colitis. However, recommendations slightly differ among scientific societies [1,32,33].

Over the past decade, the major changes in surveillance involved techniques rather than time intervals. Conventional endoscopic evaluation, including surveillance endoscopy, used to be performed in white-light (WL), meaning that the image is acquired after the mucosa is illuminated by a white light source. The picture is then digitally processed and presented on a screen [34]. The resolution of the image is one of the main factors determining its overall quality. IBD surveillance endoscopy with standard definition (SD) white light is limited by a significant lesion miss-rate (up to around 25%), especially for small and flat lesions [7]. For this reason, previous guidelines recommended taking 4 random biopsies (one per quadrant) every 10 cm along the whole colon [35,36] to increase the chance of detecting dysplasia. In addition, guidelines recommend using chromoendoscopy, which implies spraying a dye (methylene blue or indigo carmine) to enhance the features of the mucosa suspicious of dysplasia and facilitate recognition [37]. In recent years, the introduction of higher resolution endoscopes (HD) coupled with HD monitors (with a vertical resolution of 1080 pixels), provided sharper images [38] (Figure 1). The improvement in resolution allowed for better visualization of areas of suspected dysplasia and an easier targeting of biopsies [39]. The introduction of HD scopes roughly coincided with that of virtual chromoendoscopy (VCE) [40,41]. The 2015 SCENIC international consensus clarified the evidence and recommendations on IBD surveillance [32]. Experts supported the use of HD instruments over SD for surveillance endoscopy, due to the higher dysplasia and neoplasia detection rates associated with HD imaging. Guidelines also endorsed the use of chromoendoscopy (dye or dye-less), since it outperforms SD-WL in the context of surveillance [8].

Whether to prefer dye or dye-less (virtual) chromoendoscopy, current evidence remains not conclusive. A recent meta-analysis of 11 randomized controlled trials (6 in UC, 5 in both UC and CD) with a total of 1328 patients, comparing dye and virtual chromoendoscopy (NBI, FICE, i-Scan) did not find significant differences in detection of dysplasia [42]. In light of this evidence, scientific organizations gradually modified their recommendation, recognizing VCE as an acceptable alternative to dye spray [37,43].

Furthermore, the role of random biopsies has been questioned after the introduction of HD instruments, since dysplasia detected on random biopsies represent a small minority, when compared to dysplasia found in targeted biopsies [9]. Scientific societies, such as the European Crohn’s and Colitis Organization (ECCO), endorse the use of targeted biopsies, provided chromoendoscopy is used, even though some authors still also support adding random biopsies in high-risk patients (i.e., personal history of neoplasia, concomitant PSC, tubular appearing colon), in spite of the low diagnostic yield [1,10]. In the discussion over segmental random biopsies, it should also be noted that risks associated with biopsies, although very low, are not zero, and performing tens of biopsies in the same patient can ultimately cause bleeding.

Finally, a multicenter multi operator randomized controlled trial (VIRTUOSO) compared the performance of HD VCE and HD WL for the detection of colonic neoplasia in 188 IBD patients at risk of cancer (129 UC, 57 Crohn’s colitis, 2 concurrent PSC), not finding any significant difference between the two techniques (HD WLE neoplasia detection rate = 23.4%, HD virtual chromoendoscopy neoplasia detection rate = 14.9%; *p* = 0.14). These results suggest that HD itself might overcome the need for chromoendoscopy. In addition, the trial confirmed the negligible diagnostic gain of quadratic random biopsies (1 low grade dysplasia was detected out of 6751 random biopsies, and all other neoplasia were found on targeted biopsies) [11].

## 4. Endoscopy in IBD: Techniques

### 4.1. Chromoendoscopy

Dye chromoendoscopy consists in the application of contrast dye to the mucosa via the endoscope channel (Figure 1). Dyes used in IBD can either be absorptive (also known as vital) or non-absorptive [44]. The two most commonly used products are methylene blue, an absorptive stain that penetrates inside the cells, and indigo carmine, a non-absorptive dye that improves the definition of the mucosa by pooling in the irregularities of the epithelium [44]. The employment of the two aforementioned stains allows practitioners to enhance areas of abnormalities and better define the extension and margins of suspect lesions [12] without carrying any safety risk. Such areas can be better targeted by biopsies for pathological evaluation, and indeed, several studies report significantly higher dysplasia detection with targeted biopsies compared to random ones in WL endoscopy [8,13].

Further technical advancements led to the development of dye-less chromoendoscopy (Figure 1). This technology obviates the need for spraying liquid dye, overcoming some of its limitations, in particular the longer procedure time and uneven staining [45]. This is achieved through the optical and digital filtering of selected light wavelengths to enhance certain features of the mucosal surface such as the vascular pattern or erosions. Different systems of virtual chromoendoscopy exist, including Narrow-band imaging (NBI) (Olympus, Tokyo, Japan), compound-band imaging (CBI) (Aohua Photoelectricity, Shanghai, China), Fujifilm intelligent color enhancement (FICE), blue light imaging (BLI), linked color imaging, (LCI) (Fujifilm, Tokyo, Japan) and i-scan (Pentax, Tokyo, Japan) [14,15,45].

### 4.2. Video Capsule Endoscopy

Video capsule (CE) can evaluate the whole gastrointestinal tract including the small bowels, beyond the reach of conventional endoscopy, with higher sensitivity for small findings compared to bowel ultrasound, entero-CT-scan (CTE) and entero-magnetic resonance (MRE) [16,17] (Figure 1). Suspected or established CD in the small bowel are two indications for CE [46], and the main scores used for the quantification of disease activity in CD are the Lewis score (LS) [46] and the Capsule Endoscopy Crohn’s Disease Activity Index (CECDAI or Niv score) (Table 4), which grade the inflammation, extension and presence of strictures [47].

A prospective, blinded study of 93 suspected CD patients evaluated the diagnostic accuracy of CE, magnetic resonance enterography and CT enterography as compared to the gold standard, ileocolonoscopy [17]. The sensitivity and specificity for diagnosis of CD in the terminal ileum was 100% and 91% by CE, superior to MRE (81% and 86%, respectively) and CTE (76% and 85%, respectively), thus supporting the use of CE as a first line approach for detection of small bowel disease beyond the reach of ileocolonoscopy [17].

The diagnosis of postsurgical CD recurrence is another application of CE in IBD. In a study by Beltran et al., CE was superior to colonoscopy for the detection of CD recurrence in the neoileum and the rest of the bowels, as well as better tolerated [48]. Furthermore, because IBD remains unclassified (IBD-U) in up to 10–15% of cases after conventional colonoscopy and histology [49], and in 30% of these patients CD will be diagnosed at later stage, there is interest in the early detection of lesions in the small bowels that could improve correct diagnosis [50]. Different studies evaluated CE for the improvement of IBD-U diagnosis [51,52]. Mehdizadeh et al. detected findings consistent with CD in 15.8% of 20 patients previously diagnosed with UC or IBD-U who underwent CE [51]. In a similar study, CE identified CD in 5 patients out of 30 classified as IBD-U. However, in the same study, negative CE could not exclude CD. Indeed, in 6 out of 25 CE-negative patients, CD was diagnosed on a subsequent ileocolonoscopy with biopsies [52].

#### 4.2.1. Risk of Capsule Retention

The ICCE consensus defines “capsule retention” as the capsule remaining in the gastrointestinal tract more than two weeks or when endoscopic, surgical or medical intervention is required to remove it [18]. This event is generally asymptomatic, although few patients complain of partial or complete intestinal occlusion [53]. The risk of capsule retention is around 1.5% in suspected CD and 5–13% in known CD [54]. To avoid it, CE is contraindicated in patients with known bowel strictures or swallowing disorders and a history of bowel obstruction. In addition, recent abdominal surgery is a relative contraindication [55]. To limit capsule retention, a dissolvable patency capsule can be used before the CE. The dissolution starts after 30 h. When the patency capsule is successfully excreted or not detectable on radiography in the small bowel at 30 h post ingestion, it is usually safe to perform the diagnostic CE [56].

#### 4.2.2. AI for Capsule Endoscopy

Reviewing CE recordings is time-consuming and challenging. An experienced endoscopist needs to watch hours of videos searching for findings visible only in a few seconds, and hence easily missed. In fact, the main limitation of capsule endoscopy is the miss rate for solitary small bowel lesions (11%) [57]. To speed up this task and improve detection, several promising AI-powered systems have been developed. Aoki et al. trained a convolutional neural network (CNN) model to detect CD ulcer or erosions using in 5360 CE images [58]. The system was validated in a separate cohort of 10,440 images, 440 of which included pathologic findings. The machine completed the assessment in little less than 4 min with a sensitivity of 88%, specificity 99% and an overall AUROC of 0.99. Another CNN model based on 17,640 CE images from 49 patients (7391 images with mucosal ulcers and 10,249 images of normal mucosa) reached similarly high performance with an AUROC of 0.99 and accuracy higher than 95% [59]. While the algorithm will continue to improve detection, a present application of AI is the initial screening of a CE video. Computer tools can highlight frames with findings in order to expedite physicians’ assessment. Supporting this application, a study comparing review times showed that the implementation of AI systems reduced reading time from 12.2 min to 3.1 for experienced examiners and from 20.7 to 5.2 for trainees, without affecting the overall accuracy [60].

### 4.3. Molecular Imaging

Molecular imaging endoscopy aims to identify the presence of specific molecular targets in the gastrointestinal tract [19] (Figure 1). This requires exogenous fluorescent agents, such as labeled peptides or antibodies, to be applied topically or systemically. Once the label binds to the specific target (i.e., surface molecules), it serves as a molecular beacon and can be detected through fluoroscopy or confocal laser endomicroscopy (CLE) [19]. This allows the recognition of cellular or biochemical alterations of the mucosa both in vivo and ex vivo [61]. Several studies on molecular imaging have been carried out ex vivo and in vivo, in animal models and subsequently in clinical trials, showing promising results for application in the context of bowel inflammation and cancer [62].

In a phase I trial, Atreya et al. predicted clinical response to anti-TNF therapy in CD through molecular imaging [63]. With confocal laser endomicroscopy and the use of fluorescent labeled anti-TNF antibodies, they assessed the expression of membrane-bound TNF (mTNF) on intestinal cells of 25 CD patients about to start treatment with adalimumab. After 12 weeks, they observed a significantly higher response rate to adalimumab in patients with high expression of mTNF compared to patients with lower levels of mTNF (92% vs. 15%, *p* < 0.001). Furthermore, in patients with high mTNF expression, the response to treatment was sustained over a longer period of time and associated with mucosal healing at follow-up endoscopy.

In another pilot study by Rath et al., the response to vedolizumab in five CD patients with previous failure to anti-TNF was predicted in a similar way [64]. Fluorescent antibodies directed against α_4_β_7_ integrin were applied topically, and then ex vivo confocal microscopy was used to detect them and estimate integrin expression. No response to vedolizumab was observed in the three patients that had no α_4_β_7_ integrin mucosal expression, while the other two patients did show clinical response to treatment. In a similar fashion, Iacucci et al. showed how ex vivo molecular imaging with CLE could predict response to anti-TNF therapy in 29 patients with CD and UC [65]. Altogether, such evidence supports a future role of molecular imaging in treatment optimization.

Applications of molecular imaging are not limited to the prediction of treatment response. Distinguishing CD and UC can be challenging, and as mentioned before, up to 10% of IBD cases do not reach a conclusive diagnosis [66]. A study by Yantiss et al. showed how DAS-1/CG-3 molecular staining and ex vivo histopathological assessment proved effective in the differential diagnosis between UC, CD and other inflammatory conditions of the colon [67]. By the same token, in the future endoscopic molecular imaging may help distinguish between similar conditions identifying disease-specific features.

Finally, molecular imaging may have a role also in the context of dysplasia and cancer detection. Recently, in a mouse model of colitis-associated cancer, Mitsunaga et al. studied the expression of gamma-glutamiltranspeptidase (GGT), an enzyme associated with malignancy: Through an enzymatically activatable probe (gGlu-HMRG) applied topically, after just 5 min, it was possible to detect fluorescent areas, which all proved to harbor dysplasia or cancer on subsequent pathological examination [68]. When further developed, molecular imaging may represent a valuable technique for cancer surveillance and early diagnosis in patients with IBD. However, it is important to remember that allergic reactions after the injection of fluorescent contrasts, albeit rare, have been described [61].

### 4.4. Endocytoscopy

The endocytoscope (EC; Olympus Corp., Tokyo, Japan) is an optical microscope endoscope that, in addition to conventional WL and VCE, allows real-time ultra-magnified observation (Figure 1). Endocytoscopy uses a high-power fixed-focus lens, either incorporated into the endoscope or in a separate probe, to deliver up to × 1440 magnification. This allows, in vivo, a microscopic visualization only seen with CLE (confocal laser endomicroscopy) or ex vivo through conventional microscopy. However, unlike CLE, endocytoscopy does not require additional video processors or intravenous contrast, is relatively easy to perform and carries no additional risks [69]. First a mucolytic agent (N-acetylcysteine 10%) is sprayed to clean the area, then methylene blue 1% is applied to stain the nuclei. A second dye, crystal violet 0.1%, can be added to stain the cytoplasm. Afterwards, the scope is advanced to place the lens in contact with the mucosal surface and magnification is adjusted [69]. At such magnification, features such as crypt architecture, cellular infiltration and alterations in microvessels become visible. These mucosal changes can be used as a surrogate of histologic activity. A preliminary study confirmed that endocytoscopic evaluation of UC activity, graded with a dedicated score (ECS), had a stronger correlation with histology compared to normal endoscopic scores such as MES or UCEIS [70]. Another pivotal study showed how endocytoscopy could predict disease outcome by assessing the depletion of goblet cells [71]. Goblet cells are known to decrease in the presence of inflammation, but thus far, their assessment was only possible with conventional microscopy or CLE. In particular, the authors used the number of Goblet cells seen at endocytoscopy to stratify the risk of relapse in a large population of UC patients in endoscopic remission (MES 0) [71].

Endocytoscopy also holds promise in the field of colorectal lesions. Cellular-level magnification can help distinguish adenomas from malignant lesions and guide management [20]. A CAD system (EndoBrain^®^) has been successfully developed to support the distinction of neoplastic from non-neoplastic polyps visualized in endocytoscopy [72,73]. In the setting of IBD-associated dysplasia, evidence is still scant, but there have been encouraging case reports suggesting cross-field applicability [74]. It is reasonable to expect that similar CADs will also be developed for IBD-associated dysplasia in the near future. Overall, the use of endocytoscopy in IBD is still in its early days but has the potential to close the gap between endoscopic and histologic assessments, challenging the need for biopsies.

### 4.5. Artificial Intelligence

Artificial intelligence (AI) and computer-assisted diagnosis (CAD) systems are increasingly implemented in endoscopy, and IBD endoscopy is no exception. Over the last years, different groups have developed machine learning algorithms to assess disease activity in frames and videos of colonoscopies (Figure 1). In a pilot study of 29 UC patients and 6 healthy controls, an algorithm was trained to integrate the data of pixels’ colors, the red channel of the red-green-blue pixel value, with the recognition of vascular patterns. The score generated by this system (Red Density^®^) showed a strong correlation with both endoscopic (MES r = 0.76 and UCEIS r = 0.74) as well as histologic scores (RHI r = 0.74) [75]. To validate RedDensity^®^, a larger multicenter study (PROCEED) is ongoing.

Takenaka et al. used big data to develop an endoscopy AI for UC disease assessment. They trained and validated a deep neural network (DNN) algorithm using 40,758 colonoscopy images and 6885 biopsy results from 2012 UC patients. In each given frame, the algorithm predicted endoscopic remission with 93% sensitivity and 88% specificity compared to the endoscopist [76]. The DNN also had an extremely high intraclass correlation coefficient (0.917; 95% CI 0.911–0.921), indicating high agreement between human and artificial scoring. Finally, the algorithm produced a similarly accurate prediction when evaluating histologic remission, with 92% sensitivity and 93% specificity. The same group recently perfected the algorithm to assess disease activity directly on videos, not just on frames. This was assessed in a large prospective study including 770 patients and 900 biopsy specimens. In this setting, the CAD system had a sensitivity of 97.9% and a specificity of 94.6% for predicting histological remission [77].

Another promising machine learning algorithm, recently presented by Byrne and colleagues, leveraged a large number of endoscopy frames (>33.000) to improve the efficiency and accuracy of the scoring process. This model predicted both MES and UCEIS, with a mean absolute error between machine and human of 0.30 and 0.72 for MES and UCEIS, respectively [78].

The performance of computers does not fluctuate with tiredness or stress, thus improving human reliability and making up for endoscopist distraction. More importantly, AI results can be accurately reproduced. This represents a unique opportunity to standardize the assessment of disease activity, which suffers from high interobserver variability. For example, central reading in clinical trials could be simplified and expedited using the same machine learning algorithm [79].

Finally, as CAD systems evolve to detect ever more subtle changes, the gap between endoscopy and histology narrows. Because endoscopy can assess larger areas of mucosa, in the future, enhanced endoscopy might reduce the need for biopsies. In this sense, AI models have also been successfully developed to interpret ultra-magnified imaging from endocytoscopy. In a recent study of 145 UC patients in clinical remission, Maeda and colleagues, accurately stratified the risk of relapse through an AI system developed on endocytoscopy images [80].

## 5. Discussion

In IBD, as interest shifts towards objective measures of disease activity, the importance of endoscopy grows. At the same time, numerous technological advances are reducing the gap with histology, increasingly considered the new reference standard. VCE, CLE and endocytoscopy converge towards a deeper characterization of the mucosa, paving the way to a more comprehensive endoscopic assessment. The clinical implication of a deeper disease characterization is becoming ever more relevant given the expansion of the therapeutic armamentarium. Until recently, the lack of medical options limited the change of treatment, thus confining stringent therapeutic targets, such as histologic remission, to mainly prognostic factors. At present, with a growing number of approved biologics and advanced small molecules for both CD and UC, physicians have more room for treatment adjustments and can afford a lower threshold for switching medications. Modern endoscopy will play a crucial role in guiding this process. Standardization is among the main challenges that lay ahead. Advanced endoscopy technologies have great potential though their application remain limited by high inter-observer variability. It is realistic that CAD systems will soon standardize complex evaluations such as grading inflammation. AI is also contributing to the simplification of complex image analysis such as endocytoscopy and pCLE, partially overcoming their long learning curve. More broadly, AI systems reliably supporting clinicians in the interpretation of findings could reduce the need for training, serving as supervision. Innovation will also affect dysplasia detection in IBD. Computer systems that detect adenomatous polyps are widely available, and it is reasonable that similar tools will soon be developed for IBD-associated dysplasia as well.

Despite the advances, patient acceptance remains one of the main constraints of endoscopy. Video capsule is more tolerated than conventional endoscopy and could potentially be performed out of the hospital without medical supervision. AI software are dramatically reducing the time needed to review capsule videos while enhancing the detection of findings. Such advances might shift the balance in favor of a more common use of CE, particularly in settings of limited access to medical facilities, such as in the recent pandemic.

Finally, pioneering studies on molecular endoscopy are paving the way to personalized medicine. As the number and cost of available medication increases, tools to predict response and guide choice of treatment are in great need. Assessing mucosal expression of biomarkers represents a promising approach not only to select treatment but more broadly to characterize tissue. In a similar way, molecular characterization has improved our understanding of cancer and its treatment.

Innovations in AI for disease assessment and surveillance are expected to impact a large number of IBD patients in the next years, improving quality of care. We foresee a widespread adoption of CAD systems in capsule endoscopy reviewing, inflammation assessment and, soon, cancer surveillance. More sophisticated technologies such as endocytoscope and pCLE are likely to gain ground although, their adoption out of tertiary centers at present remains limited by high costs and need for special expertise. Finally, molecular imaging, while extremely promising from a theoretical perspective, needs more validation and cost effectiveness analysis before being proposed in clinical practice.

New technologies also carry new challenges: first of all, costs. Medical care is already expensive, and access is often limited by budget constraints. Thus, new equipment could potentially widen the gap between resource-rich and poor settings. Secondly, machine learning algorithms are dependent on the population they are trained on, which tends to over-represent white Westerners to the detriment of other races and ethnicities. From a regulatory perspective, as interest in big data increases, so do privacy concerns. Finally, when computer algorithms will gain a more active role in medical decision making, legal implications will arise, requiring an update of the legislation.

In conclusion, IBD endoscopy is evolving to encompass new aspects of disease assessment. The coming years are likely to witness a remarkable upgrade in the role of endoscopy in the care of UC and CD.

## Figures and Tables

**Table 1:** Rutgeerts’ score.

Rutgeerts Grade	Endoscopic Finding
i0	Absence of lesions in the terminal ileum
i1	Up to 5 anastomotic aphtous lesions in the terminal ileum
i2	Over 5 aphtous lesions with unaffected mucosa between lesions, skip areas of larger lesions or ulcers no larger than 10 mm limited to the ileo-colonic anastomosis
i3	Diffuse aphtous ileal flogosis with inflamed mucosa between aphtae
i4	Diffuse inflammation and associated larger lesions: ulcers larger than 10 mm, cobble/nodules or narrowing/stenosis

**Table 2:** UCEIS score.

Descriptor	Score	Definition
Vascular pattern	Normal (0) Patchy obliteration (1) Obliterated (2)	Normal vascular pattern with arborization of capillaries clearly defined or with blurring or patchy loss of capillary margins Patchy obliteration of vascular pattern Complete obliteration of vascular pattern
Bleeding	None (0) Mucosal (1) Luminal mild (2) Luminal moderator severe (3)	No visible blood Some spots or streaks of coagulated blood on the surface of the mucosa ahead of the scope, which can be washed away Some free liquid blood in the lumen Frank blood in the lumen ahead of endoscope or visible oozing from mucosa after washing intraluminal blood or visible oozing from a hemorrhagic mucosa
Erosions and ulcers	None (0) Erosions (1) Superficial ulcer (2) Deep ulcer (3)	Normal mucosa, no visible erosions or ulcers Tiny (≤5 mm) defects in the mucosa, which are discrete fibrin-covered ulcers in comparison with erosions, but remain superficial Larger (>5 mm) defects in the mucosa, which are discrete fibrin-covered ulcers in comparison with erosions, but remain superficial Deeper excavated defects in the mucosa, with a slightly raised edge

**Table 3:** UCCIS score.

Lesion	Score	Definition
Vascular pattern	0	Normal, clear vascular pattern
	1	Partially visible vascular pattern
	2	Complete loss of vascular patter
Granularity	0	Normal, smooth and glistening
	1	Fine
	2	Coarse
Ulceration	0	Normal, no erosion or ulcer
	1	Erosions or pinpoint ulcerations
	2	Numerous shallow ulcers with mucopus
	3	Deep, excavated ulcerations
	4	Diffusely ulcerated with >30% involvement
Bleeding friability	0	Normal, no bleeding, no fraibility
	1	Friable, Bleeding to light touch
	2	Spontaneous bleeding
Grading of SAES and GAES (4-point scale)	0	Normal/quiescent: visible vascular pattern with no bleeding, erosions, ulcers, or friability
	1	Mild: eritherma, decreased or loss of vascular pattern, fine granularity, but no fraibility or spontaneous bleeding
	2	Moderate: fraibility with bleeding to light touch, coarse granularity, erosions, or pintpoint ulcerations
	3	Severe: spontaneous bleeding or gross ulcers
GAES VAS 10-cm scale		(0) (10)
		Normal Extremely severe

**Table 4:** Lewis score and CECDAI/NIV score.

Name	Formula	Notes
Lewis Score	[(Villous parameter × extent × descriptor) + (Ulcer parameter × extent × size)] for tertile 1, 2 or 3 + (Stenosis number × ulcerated × traversed).	The total time of video capsule progression among the bowel is divided in three tertiles, and the score is calculated as the most severe tertile score plus stenosis <135 clinically insignificance 135–790 mild >790 moderate to severe damage
CECDAI or NIV	A. Inflammation score 0 = None 1 = Mild to moderate edema/hyperemia/denudation 2 = Severe edema/hyperemia/denudation 3 = Bleeding, exudate, aphthae, erosion, small ulcer (<0.5 cm) 4 = Moderate ulcer (0.5–2cm), pseudo polyp 5 = Large ulcer (>2cm) B. Extent of disease score 0 = No disease –normal examination 1 = Focal disease (single segment is involved) 2 = Patchy disease (2–3 segments are involved) 3 = Diffuse disease (more than 3 segments are involved) C. Stricture score 0 = None 1 = Single-passed 2 = Multiple-passed 3 = Obstruction (non-passage) Segmental score (proximal or distal) = (A × B) +C Total score =proximal ([A × B] + C) +distal ([A × B] + C) CEDCAI = proximal ([A × B] + C) + distal ([A × B] + C).	The score is included in the interval 0 (no damage) to 26 (severe inflammation).

## Data Availability

Not applicable.
